# A prenatal standard for fetal weight improves the prenatal diagnosis of small for gestational age fetuses in pregnancies at increased risk

**DOI:** 10.1186/s12884-022-04545-x

**Published:** 2022-03-26

**Authors:** Silvia Visentin, Ambrogio P. Londero, Ilaria Cataneo, Federica Bellussi, Ginevra Salsi, Gianluigi Pilu, Erich Cosmi

**Affiliations:** 1 Department of Woman’s and Child’s Health, Maternal-Fetal Medicine Unit, University of Padua, Via Nicolò Giustiniani, 3, 35128 Padua, Padova Italy; 2 Academic Unit of Obstetrics and Gynaecology, Department of Neuroscience, Rehabilitation, Ophthalmology, Genetics, Maternal and Infant Health, University of Genoa, Largo Rosanna Benzi, 10, 16132 Genoa, GE Italy; 3 Obstetric Unit, Department of Medical and Surgical Sciences, University of Bologna, Bologna, Italy

**Keywords:** Small for gestational age, INTERGROWTH-21st, Pre-natal growth chart, Post-natal growth chart

## Abstract

**Objective:**

Our aim was to assess diagnostic accuracy in the prediction of small for gestational age (SGA <10th centile) and fetal growth restricted (FGR) (SGA <3rd centile) fetuses using three different sonographic methods in pregnancies at increased risk of fetal growth restriction: 1) fetal abdominal circumference (AC) z-scores, 2) estimated fetal weight (EFW) z-scores according to postnatal reference standard; 3) EFW z-scores according to a prenatal reference standard.

**Methods:**

Singleton pregnancies at increased risk of fetal growth restriction seen in two university hospitals between 2014 and 2015 were studied retrospectively. EFW was calculated using formulas proposed by the INTERGROWTH-21st project and Hadlock; data derived from publications by the INTEGROWTH-twenty-first century project and Hadlock were used to calculate z-scores (AC and EFW). The accuracy of different methods was calculated and compared.

**Results:**

The study group included 406 patients. Prenatal standard EFW z-scores derived from INTERGROWTH-21st project and Hadlock and co-workers performed similarly and were more accurate in identifying SGA infants than using AC z-scores or a postnatal reference standard. The subgroups analysis demonstrated that EFW prenatal standard was more or similarly accurate compared to other methods across all subgroups, defined by gestational age and birth weight.

**Conclusions:**

Prenatal standard EFW z-scores derived from either INTERGROWTH-21 st project or Hadlock and co-workers publications demonstrated a statistically significant advantage over other biometric methods in the diagnosis of SGA fetuses.

## Synopsis

Prenatal growth chart z-score had better diagnostic performances than post-natal growth chart z-score in identifying small for gestational age fetuses in pregnancies at increased risk of growth restriction, and estimated fetal weight z-scores were more accurate than abdominal circumference z-score in the prediction of small for gestational age.

## Highlights - Whats this paper adds

- Prenatal based fetal weight reference z-scores were more accurate in identifying small neonates than post-natal fetal weight reference z-scores and abdominal circumference z-scores;
- The INTEGROWTH-21st approach, consisting of one formula to calculate fetal weight and of a prenatal reference standard performed better or similarly than other methods across all subgroups defined by gestational age and neonatal size.

## Introduction

Identifying small for gestational age (SGA) fetuses has a paramount value in contemporary obstetric management [1–7]. At present, the cornerstone for the diagnosis is sonographic biometry, that despite recent improvements in technology, remains imprecise [4, 5, 8, 9]. The limitations of ultrasound are well-known and have been previously discussed at length. The consensus is that the measurement of the abdominal circumference (AC) and the estimated fetal weight (EFW) are anyhow the best predictors, and that they provide very similar results and are, in essence, interchangeable [1, 2, 9]. Indeed, it has been suggested that calculating the EFW is unnecessary for diagnostic purposes, as it is more complex and cumbersome than obtaining an AC measurement without improving the accuracy [8, 9]. However, in most of the available studies, EFW was compared with standards obtained from neonates. There is evidence indicating that preterm newborns may be smaller than fetuses in ongoing pregnancies [10–12]. Indeed, prematurity is frequently associated with obstetric complications, including placental insufficiency [13]. Underestimation of the normal range would result in an obvious bias. Theoretically, a reference standard of fetal weight obtained from sonographic measurements of fetuses in ongoing pregnancies would be preferable than using birthweights of premature infants. Thus far, this has been prevented by the paucity of studies reporting fetal standards [10], which has been further exasperated by the prevailing concept that ethnicity is a major determinant of fetal growth and that clinicians should favor standards derived from their own population [14]. The INTERGROWTH-21st (IG-21) project has recently challenged this view, providing evidence that fetal size is unrelated to ethnicity [15–17]. Within this project, an international standard of fetal weight based upon prospective sonographic measurements, as well as a novel formula to calculate the EFW, have been made available [11].

The aim of our study was to evaluate and compare the diagnostic accuracy in the prediction of SGA <10th centile and FGR (SGA <3rd centile) fetuses of the IG-21 prenatal standard of EFW versus neonatal standards and AC measurements in a group of pregnancies at increased risk of growth restriction.

## Materials and methods

The study group was described in detail in a previous publication [8]. A cohort was created from the databases of Obstetrics and Gynecology departments from the universities of Bologna and Padua, including all singleton pregnancies with an increased risk of intrauterine growth restriction that had undergone at least one ultrasound examination between 24 and 36 weeks’ gestation in the period 2013–2015. Only one scan for each patient was selected in each analysis, the one closer to the delivery date in the gestational period analyzed. In our setting were usually considered at increased risk of intrauterine growth restriction development the pregnancies with the following characteristics: abnormal maternal Doppler at anomaly scan, previous history of small for gestational age newborns, pre-pregnancy diabetes, gestational diabetes, pre-gestational or gestational hypertension, rheumatological or immunological disorders, smoking during pregnancy, and pregnancies referred from other centers as at increased risk of intrauterine growth restriction development. We excluded: twin pregnancies and newborns with congenital malformations. The sonograms and clinical information (maternal and neonatal) data were gathered from the two databases. This clinical audit of fully anonymized data without an experimental design was conducted according to national regulation and was approved by the local ethics committee (Comitato Etico per la Sperimentazione Clinica della Provincia di Padova). Fetal biometry was performed in each case as previously described, AC z-scores were calculated from the IG-21 standard [15], and the EFW was calculated using the formulas proposed by the IG-21 project [11] and by Hadlock and co-workers. Neonates were considered SGA if the birth weight was less than the 10th centile according to the IG-21 postnatal standard, and fetal growth restriction (FGR) was considered as SGA less than the 3rd centile [16]. Three methods for the prenatal diagnosis of SGA neonates were considered and compared: a) AC z-scores derived from the IG-21 project [15]; b) EFW calculated with the formula proposed by the IG-21 project [11] and stratified in z-scores derived from the postnatal IG-21 standard [12, 16] (EFW postnatal standard); c) EFW calculated with the formula and stratified with the prenatal standards proposed by Hadlock and co-workers [19] (EFW prenatal standard Hadlock) and by the IG-21 project [11] (EFW prenatal standard IG-21). Subgroup analyses were performed to evaluate the ability of the different methods to predict a birthweight <10th and 3rd centile and in different gestational age periods.

The R program (version 3.4.1, R Foundation for Statistical Computing, Vienna, Austria, http://www.R- project.org/) was used for statistical analysis. The *p*-value < 0.05 was defined as significant considering a two-sided alternative hypothesis. Based on the preliminary dataset, the sample size was established a priori to find a significant difference of at least 5% between two curves to predict SGA <10th centile with a significant level of 0.05 and an 80% power. The normality of variable distribution was assessed using the Kolmogorov-Smirnoff test. Data were presented by mean (standard deviation), median and interquartile range (IQR), percentage and absolute values, and a specified reference value (e.g., area under the curve (AUC)) and 95% confidence interval (CI). During the analysis, where appropriate, we used the following statistical tests: in case of continuous variables, Student’s t-test or Wilcoxon test; in case of categorical variables, Fisher’s exact test or chi-square. The prediction accuracy of fetal biometry to forecast SGA <10th percentile and FGR (SGA <3rd percentile) was assessed by the area under the receiver operating characteristics (ROC) curves with the relative 95% CI and DeLong’s test. Besides, the sensitivity at a 10% false-positive rate (FPR) was calculated.

## Results

We retrospectively collected 995 ultrasound examinations from 406 singleton pregnancies, performed between 24 and 36 weeks’ gestation. Among the selected singleton pregnancies, 93 (22.91%) delivered SGA newborns <10th weight percentile and 35 (8.62%) FGR (SGA newborns <3rd weight percentile).

The characteristics of the population are reported in Table 1. The median age of women was 34 years (IQR 30–38), the median BMI was 26 kg/m2 (IQR 23–29), and the median gestational age at delivery was 38 weeks (IQR 37–39). Newborns were males in 47.54, and 60.59% of women were nulliparous.

**Table 1:** Characteristics of the population. Data are reported as median and interquartile range (IQR) or as absolute values and percentage

Mother age (years)	34 (30–38)
Pre-pregnancy BMI (Kg/m^2^)	26 (23–29)
Race	
White	80.3% (326/406)
Black	15.76% (64/406)
Asian	3.94% (16/406)
Nulliparity	60.59% (246/406)
Gestational age at birth (weeks)	38 (37–39)
Mode of delivery	
Spontaneous vaginal delivery	41.87% (170/406)
Operative vaginal delivery	4.68% (19/406)
Cesarean section	53.45% (217/406)
Neonatal weight (grams)	2800 (2450–3178)
Neonatal male sex	47.54% (193/406)

Table 2 reports the accuracy of the different methods to predict SGA newborns. We found that EFW prenatal standards according to Hadlock and IG-21 performed similarly and were significantly more accurate than AC and EFW postnatal standards.

**Table 2:** Accuracy analyses to predict SGA newborns. *P*-value refers to DeLong’s test (AC, abdominal circumference measurement stratified according to Papageorghiou et al. [15]; EFW postnatal standard, estimated fetal weight calculated with the formula proposed by Stirnemann et al. [11] and stratified according to the standard reported by Villar et al. [12, 16]; EFW prenatal standard IG-21, estimated fetal weight calculated with the formula proposed by Stirnemann et al. [11] and stratified according to the standard reported by Stirnemann et al. [11]; EFW prenatal standard Hadlock, estimated fetal weight calculated with the formula proposed by Hadlock et al. [18] and stratified according to the standard reported by Hadlock et al. [19]). It is reported, also the sensitivity at 10% false positive rate (FPR)

Birth-weight centile	Variable	Area under ROC curve % (95% CI)	Sensitivity at 10% FPR % (95% CI)	*p* value		
				AC	EFW postnatal standard	EFW prenatal standard IG-21
< 10th	AC	84.91 (82.33–87.49)	53.18 (43.18–62.05)	–		
	EFW postnatal standard	83.07 (80.37–85.77)	46.82 (35.91–56.82)	< 0.05	–	
	EFW prenatal standard IG-21	86.52 (84.15–88.90)	54.55 (46.82–63.18)	< 0.05	< 0.05	–
	EFW prenatal standard Hadlock	87.27 (84.96–89.58)	55.91 (47.27–63.64)	< 0.05	< 0.05	0.190
< 3rd	AC	88.81 (85.67–91.96)	56.10 (43.90–68.97)	–		
	EFW postnatal standard	86.78 (83.25–90.31)	56.10 (45.12–68.29)	< 0.05	–	
	EFW prenatal standard IG-21	89.85 (86.77–92.93)	64.63 (50.00–78.05)	< 0.05	< 0.05	–
	EFW prenatal standard Hadlock	89.51 (86.35–92.66)	59.76 (46.34–71.95)	0.344	< 0.05	0.562

**Fig. 1:**
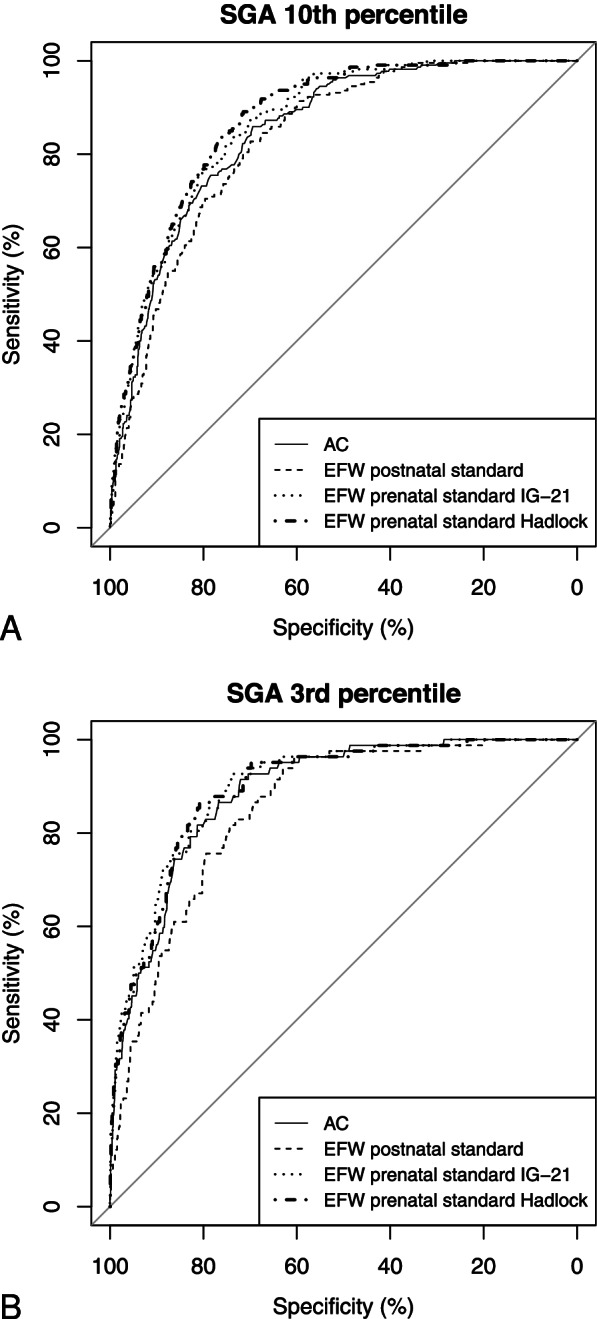
Receiver operator characteristic curves comparing the diagnostic accuracy of abdominal circumference [15]; estimated fetal weight calculated using the INTERGROWTH-21st project formula [11] stratified according to a postnatal reference standard [12, 16] (EFW postnatal standard); estimated fetal weight calculated using the INTERGROWTH-21st project formula [11] statified according to the IG-21 prenatal reference standard [11] (EFW prenatal standard IG-21); estimated fetal weight calculated using the Hadlock and co-workers formula statified according the pretnatal reference standard proposed in the same publication [18, 19] (EFW prenatal standard Hadlock). Panel A) refers to SGA <10th percentile. Panel B) refers to FGR (SGA <3rd percentile)

The IG-21 prenatal standard was more accurate in identifying both fetuses <10th and 3rd centiles than AC and EFW postnatal standards. EFW, according to Hadlock and co-workers, was more accurate than AC in identifying SGA < 10th centiles but performed similarly for fetuses <3rd centile (FGR) (Fig. 1A and B).

The accuracy of SGA prediction by stratifying for gestational age at ultrasound examination is summarized in Table 3. The Hadlock and IG-21 EFW prenatal standards performed similarly or better than other biometric methods in each time window.

**Table 3:** Accuracy analyses to predict SGA newborns, in this analysis prediction accuracy was stratified for gestational age at ultrasound examination. P-value refers to DeLong’s test. (AC, abdominal circumference measurement stratified according to Papageorghiou et al. [15]; EFW postnatal standard, estimated fetal weight calculated with the formula proposed by Stirnemann et al. [11] and stratified according to the standard reported by Villar et al. [12, 16]; EFW prenatal standard IG-21, estimated fetal weight calculated with the formula proposed by Stirnemann et al. [11] and stratified according to the standard reported by Stirnemann et al. [11]; EFW prenatal standard Hadlock, estimated fetal weight calculated with the formula proposed by Hadlock et al. [18] and stratified according to the standard reported by Hadlock et al. [19])

Birthweight centile (gestational age)	Variable	Area under ROC curve % (95% CI)	*p* value		
			AC	EFW postnatal standard	EFW prenatal standard IG-21
< 10th (24–27 wks)	AC	86.91 (79.91–93.91)	–		
	EFW postnatal standard	89.4 (82.94–95.8)	0.970	–	
	EFW prenatal standard IG-21	88.15(81.56–94.7)	0.191	0.312	–
	EFW prenatal standard Hadlock	89.7 (C 83.46–95.9)	0.127	< 0.05	0.262
< 3rd (24–27 wks)	AC	90.7 (81.09–100)	–		
	EFW postnatal standard	90.98 (C 80.17–100)	0.829	–	
	EFW prenatal standard IG-21	92.09 (82.41–100)	0.256	0.319	–
	EFW prenatal standard Hadlock	91.86% (81.5–100)	0.489	0.426	0.829
< 10th (28–33 wks)	AC	85.14 (81.52–88.75)	–		
	EFW postnatal standard	87.13 (83.87–90.38)	0.257	–	
	EFW prenatal standard IG-21	87.13 (83.87–90.38)	< 0.05	< 0.05	–
	EFW prenatal standard Hadlock	88.68 (85.66–91.69)	< 0.05	< 0.05	< 0.05
< 3rd (28–33 wks)	AC	89.87 (85.99–93.75)	–		
	EFW postnatal standard	89.08 (84.77–93.39)	0.350	–	
	EFW prenatal standard IG-21	90.92 (87.13–94.72)	0.077	< 0.05	–
	EFW prenatal standard Hadlock	90.67 (86.81–94.53)	0.447	0.173	0.769
< 10th (34–36 wks)	AC	84.49 (80.33–88.64)	–		
	EFW postnatal standard	85.11 (81.18–89.04)	0.362	–	
	EFW prenatal standard IG-21	85.5 (81.6–89.46)	0.082	0.363	–
	EFW prenatal standard Hadlock	84.75 (80.62–88.88)	0.841	0.763	0.449
< 3rd (34–36 wks)	AC	84.49(80.33–88.64)	–		
	EFW postnatal standard	85.11 (81.18–89.04)	0.751	–	
	EFW prenatal standard IG-21	85.53 (81.6–89.46)	0.372	0.322	–
	EFW prenatal standard Hadlock	84.75 (80.62–88.88)	0.828	0.711	0.795

## Discussion

### Principal findings

Our results confirm the hypothesis that matching the EFW against a prenatal standard improves SGA diagnosis. The Hadlock and the IG-21 prenatal standards performed better than the traditional approach using a postnatal standard. This result is in keeping with previous studies suggesting that a growth chart derived from birthweights underestimates fetal size in early gestation and hinders the diagnosis of small fetuses [10]. Furthermore, the EFW prenatal standards were more accurate than AC measurement across all birthweight subgroups. This result is at variance with previous publications suggesting the EFW and AC perform similarly [8, 9, 20] or suggesting AC performs better than EFW in some circumstances [20].

Stratification of diagnostic accuracy in different gestational age windows demonstrated that the performance of all methods was similar, with minimal differences, most likely because of the small sample size of each subgroup. In essence, the combination of the formula to calculate the EFW and the standard proposed by the Ig-21 was more or similarly accurate compared to other methods across all subgroup analyses, defined by gestational age and birth weight.

### Strengths and limitations

The main strength of our study is that, to our knowledge, it is the first to directly investigate the added value of a prenatal standard for the EFW in a large group of pregnancies at increased risk for growth restriction. Several limitations deserve consideration. First, the definition of SGA neonates is controversial. We have used the approach supported by the IG-21 project, which has provided convincing evidence that fetal growth is independent of ethnicity in normal pregnancies. Other studies suggest that customized charts, considering ethnic and parental factors, allow a more precise prediction of adverse outcomes and maybe, therefore, more in keeping with the concept of growth restriction [14, 21]. There is an ongoing debate that is not likely to find a solution soon [21–25], and the design of our study does not provide any contribution in that direction. Second, the IG-21 approach was found to have a statistically significant advantage over other methods in identifying small neonates, but the difference is relatively small. Whether this would translate into a tangible clinical improvement remains to be demonstrated.

### Generalizability

The generalization of our results is limited by our selective high-risk population sample, yielding a high prevalence of SGA and increased surveillance and attention that could have raised the sensitivity of the operators.

### Clinical implications

Our study confirms that prenatal identification of SGA neonates is imprecise [8, 9, 26]. It has been previously pointed out that the diagnosis is a clinical one and a demanding one; and that it should take into account a combination of several biometric and biophysical variables [11]. In our hands, however, stratification of the fetal weight using a prenatal standard was the most efficient parameter in pregnancies at increased risk. Furthermore, despite in the late third trimester, prenatal and postnatal charts were overlapping in their predictive efficacy in the early third trimester, prenatal standards were significantly better performers. Hence in the complex, we consider the prenatal standards the better choice. Although intergrowth 21 was significantly more predictive than AC for FGR (SGA <3rd centile), and Hadlock was not, weighing the global performance, both Hadlock and co-workers and IG-21 formulas and reference charts performed similarly. The choice of using one or the other will depend upon the preference of the practitioner (both are equivalent or superior to other methods). The IG-21 approach has, however, at least two essential advantages: it is part of a corpus of studies describing in detail prenatal and postnatal growth and provides free access to data and convenient tools (https://intergrowth21.tghn.org/). Moreover, the present study was not powered to assess the adverse neonatal outcomes. Nonetheless, we believe that future research is required to design adequately powered studies to determine the performance of different EFW charts to predict newborns’ short- and long-term outcomes.

## Conclusions

Prenatal standard EFW z-scores derived from either INTERGROWTH-21 st project or Hadlock and co-workers publications demonstrated a statistically significant advantage over AC z-scores and postnatal reference standards in diagnosing SGA fetuses.

## Data Availability

The data supporting the findings of this study are available, but restrictions apply to their circulation as they were used under license for the present study, and are therefore not publicly available. The data may nonetheless be made available by the authors on reasonable request with the permission of the Ethics Committee.

## Abbreviations

AC: Abdominal circumference
AUC: Area under the curve
BMI: Body mass index
CI: Confidence interval
EFW: Estimated fetal weight
FGR: Fetal growth restriction
FPR: false positive rate
IG-21: INTERGROWTH-21st
IQR: Interquartile range
ROC: Receiver operating characteristics curve
SGA: Small for gestational age
